# *CTNNA1*-associated retinal dystrophy: novel multimodal imaging and electrophysiology features

**DOI:** 10.1007/s10633-025-10027-0

**Published:** 2025-06-02

**Authors:** Jonathan A. Alexis, Prathiba Ramakrishnan, Matthew K. Kenworthy, Jennifer A. Thompson, Enid S. Chelva, Fred K. Chen

**Affiliations:** 1 Ocular Tissue Engineering Laboratory, Lions Eye Institute, 2 Verdun Street, Nedlands, WA Australia; 2 Australian Inherited Retinal Disease Registry and DNA Bank, Department of Medical Technology and Physics, Sir Charles Gairdner Hospital, Nedlands, WA Australia; 3 Centre for Ophthalmology and Visual Science, The University of Western Australia, Perth, WA Australia; 4 Ophthalmology, Department of Surgery, University of Melbourne, Melbourne, VIC Australia

**Keywords:** Butterfly-shaped pigment dystrophy, Macular pattern dystrophy, Microperimetry, Retinitis pigmentosa, Best’s disease

## Abstract

**Purpose:**

To describe multimodal imaging and electrophysiology features of *CTNNA1*-associated retinal dystrophy in a family with p.(Leu318Ser) substitution.

**Methods:**

Three family members including a 48-year-old male proband, his 52-year-old sister, and their 67-year-old mother, were evaluated with multimodal imaging and electrophysiology. The proband, referred with suspected Best’s disease, underwent a retinal dystrophy panel and two affected family members were target sequenced for the familial variant.

**Results:**

The NM_001903.5:c.953T > C variant in *CTNNA1* segregated with affected family members. They maintained a visual acuity of 20/25 or better throughout 2–4 years of follow-up. The proband exhibited butterfly-shaped pigment dystrophy whilst his sister had no macular lesions, and their mother had foveal pigmentary changes. All three displayed peripheral retinal reticular pigmentation with variable atrophy. Microperimetry demonstrated enlarging paracentral scotoma in the proband whilst Esterman binocular suprathreshold test showed reproducible peripheral loss in the proband’s sister. Multifocal electroretinography (ERG) confirmed central macular dysfunction in the proband. In all three, full-field ERG showed mildly delayed dark-adapted (DA) 0.01 b-wave and DA3.0 a-wave, and a light-rise of < 1.7 in one or both eyes on electro-oculography (EOG).

**Conclusions:**

*CTNNA1*-associated retinal dystrophy due to p.(Leu318Ser) has a unique peripheral retinal phenotype despite variable macular involvement. Reduced EOG light-rise and peripheral reticular pigmentation should raise suspicion of *CTNNA1* in butterfly-shaped pigment dystrophy.

**Supplementary Information:**

The online version contains supplementary material available at 10.1007/s10633-025-10027-0.

## Introduction

Butterfly-shaped pigment dystrophy is an autosomal dominant retinopathy characterised by linear foveal lesions that resemble the wings of a butterfly [1]. This phenotype is associated with heterozygous variants in peripherin-2 (*PRPH2*, OMIM #169,150), orthodenticle homeobox 2 (OTX2, OMIM #610,125) and catenin alpha-1 (*CTNNA1*, OMIM #608,970) genes. While the variable phenotype and natural history of *PRPH2*- and *OTX2*-associated retinal dystrophies have been described in large cohorts [2–5], clinical features of *CTNNA1*-associated retinal dystrophy are less well studied [6–8].

Among 26 previously reported cases from 10 families affected by dominant *CTNNA1*-associated disease (Supplementary material S1), all but three presented with foveal pigmentary lesions: a 4-year-old girl with a foveal deposit [8], a 7-year-old girl with a normal fundus [7], and a 19-year-old woman with a subtly altered foveal reflex [8]. Description of the macular lesions varied from the typical butterfly-shaped pigment dystrophy to linear-track or star-fish hyperpigmentation, “dot and halo” lesions, radial subretinal deposits, subfoveal fluid misdiagnosed as central serous retinopathy, and variable degrees of retinal pigment epithelium (RPE) atrophy [7–9]. Peripheral retinal changes characterised by drusen, atrophy, reticular lesions, and bone spicule-like pigmentation, were reported in a minority of cases but not illustrated using ultrawide-field imaging. Full-field electroretinography (ERG) was reported as normal in all 17 cases examined while 13 of 18 (72%) with electro-oculography (EOG) showed a reduced light-rise (< 1.7) [10] in one or both eyes [7–9]. Macular function assessment and detailed ERG analysis were lacking, and microperimetry data were unavailable. Short-term follow-up (≤ 3 years) showed stability in visual acuity, though subretinal deposits varied.

Herein, we describe the multimodal imaging and electrophysiology of three members from two generations of a family with the NM_001903.5:c.953T > C p.(Leu318Ser) missense variant in *CTNNA1*. Macular and peripheral retinal structure–function correlation and natural history progression were examined.

## Methods

Three members of one family (Supplementary Material S2) with *CTNNA1*-associated retinal dystrophy were recruited into the Western Australian Retinal Degeneration study. Approval for this research was obtained from the Human Ethics Office of Research Enterprise, The University of Western Australia (2021/ET000151). Each participant provided informed consent for their involvement in the research.

Ultrawide-field (UWF) images were captured using the Optos® P200DTx (Optos, California USA), while short-wave and near-infrared fundus autofluorescence (FAF) imaging and spectral-domain optical coherence tomography (OCT) were obtained using HRA2 and SPECTRALIS® HRA + OCT (Heidelberg Engineering GmbH, Heidelberg, Germany). Microperimetry was performed with the MAIA Macular Integrity Assessment System (CenterVue, Padova, Italy) using a 10–2 testing grid. To evaluate the impact of peripheral retinal changes, Esterman binocular suprathreshold test (EBST) was performed with fixation using Humphrey Field Analyzer II-i1 740 (Carl Zeiss Meditec AG, Jena, Germany). Electrophysiology was conducted using RETI-port 3.2 (Roland Consult Stasche & Finger GmbH, Brandenburg an der Havel, Germany) incorporating the International Society for Clinical Electrophysiology of Vision (ISCEV) standards for electro-oculography (EOG) and full-field, multifocal and pattern electroretinography (ERG) [10–13]. HK-Loop and skin electrodes were used for ERG and EOG respectively. Data is displayed as response waveforms and ERG component scatter plots comparing cases to healthy controls.

Genetic testing, primer designs and polymerase chain reactions (PCR) were performed at Molecular Vision Laboratory (Oregon USA). Direct testing for pathogenic variants in the genes of the MVL Vision Panel v17 (1023 genes + mitochondrial genome + copy number variation analysis) was performed by target enrichment (capture) and Next Generation Sequencing. Direct testing for the c.953 T > C p.(Leu318Ser) variant in *CTNNA1* was performed by PCR amplification and DNA Sanger sequencing in two directions. The Sanger sequencing step was performed at NeoGenomics Laboratories Inc (Texas USA). The *CTNNA1* c.953 T > C p.(Leu318Ser) variant is considered likely pathogenic per ACMG/AMP and ClinGen guidelines (PS4_MOD, PM2_SUPP, PP1_STR, PP4). ClinVar classifies it as pathogenic, while Franklin classifies it as a VUS leaning towards likely pathogenic but does not account for cases reported in the literature or this study [14–16].

### Clinical features

**Fig. 1:**
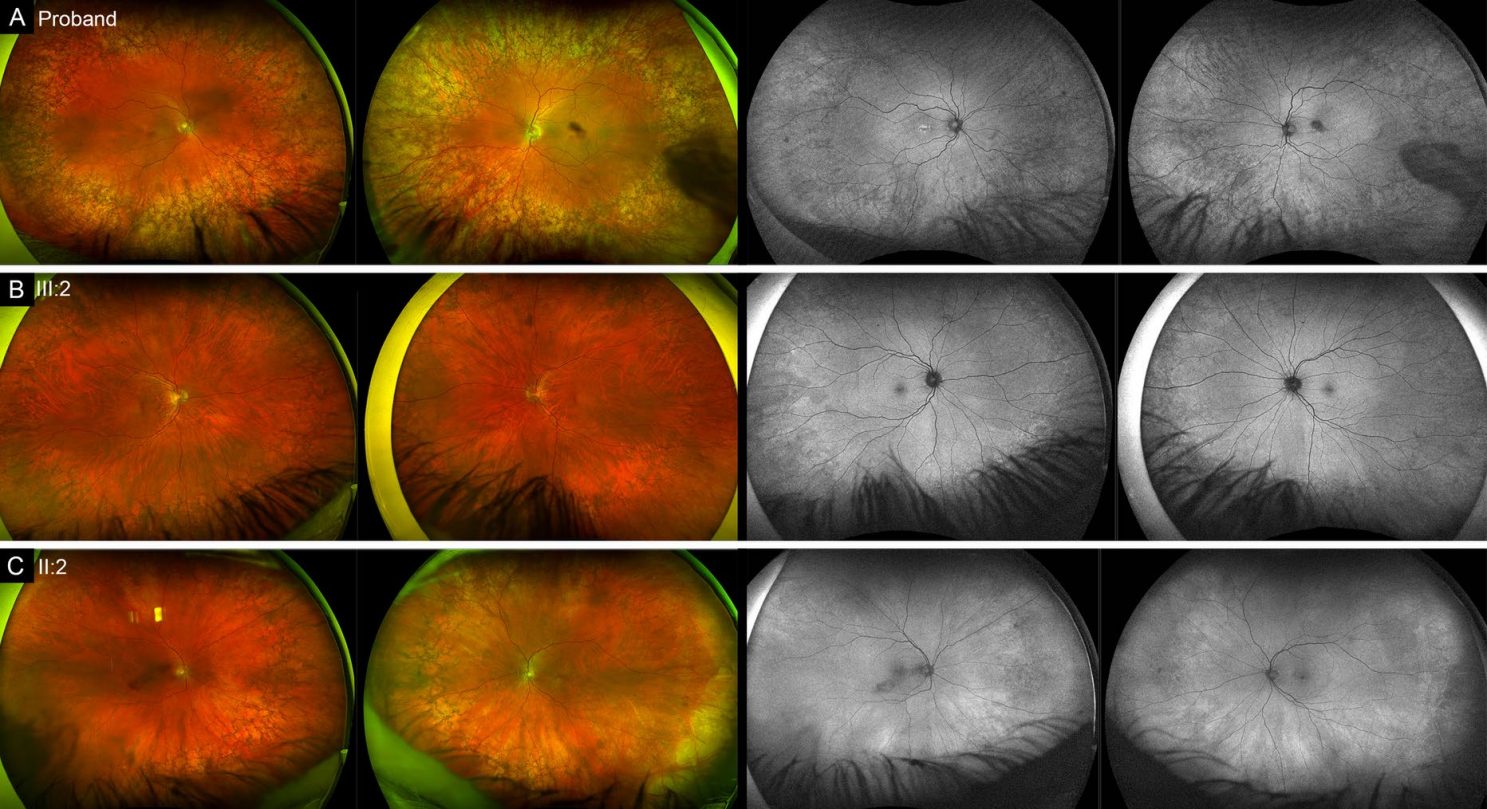
Ultrawide field pseudocolor (columns 1 and 2) and autofluorescence (columns 3 and 4) imaging of the proband (**A**), his sister (**B**) and their mother (**C**) showing the annular peripheral reticular pigmentary changes in all three patients

The proband **(III:4)**, a 48-year-old male, was referred for further assessment of suspected Best’s disease based on an abnormal EOG and a co-existing unusual peripheral pigmentary retinopathy. He had no nyctalopia but reported occasional white dots in his vision on eccentric gaze. He had a history of type 2 diabetes mellitus, ischaemic heart disease and craniosynostosis. His medications included aspirin, atorvastatin, metformin, allopurinol and carbamazepine. None of his four children aged 6 to 24 had an ocular history. His visual acuities were 20/20 in the right eye (OD) and 20/25 in the left eye (OS). Fundoscopy showed linear foveal pigmentation and an annular region of peripheral reticular hyperpigmentation (Fig. 1A). His condition remained stable over 32 months. The proband’s 52-year-old sister **(III:2)** reported no symptoms and had visual acuities of 20/20 OD and 20/16 OS. Her fundoscopy showed no macular abnormality and a similar bilateral peripheral reticular hyperpigmentation (Fig. 1B). The proband’s 67-year-old mother **(II:2)** was asymptomatic, with visual acuities of 20/25 OD and 20/20 OS. Fundoscopy revealed foveal pigmentation with the same peripheral reticular hyperpigmentation (Fig. 1C). The mother has three siblings, of which two are deceased and one is alive at age 80 years with no ocular history. The rest of the ocular examination was unremarkable except for an early posterior subcapsular cataract in the proband. A summary of the clinical findings for all three patients is shown in Supplementary Material S3.

### Multimodal imaging

**Fig. 2:**
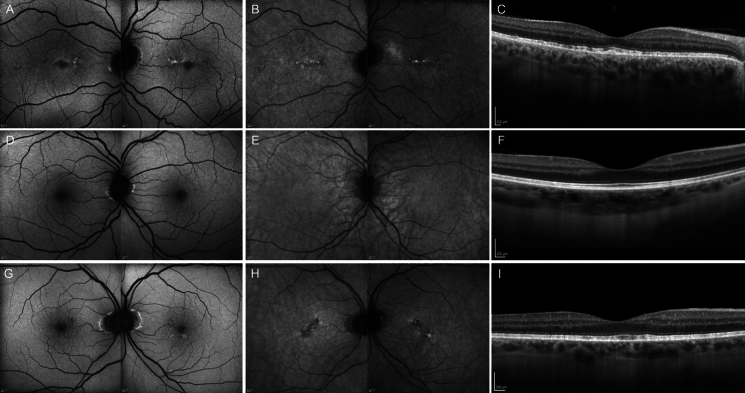
Short-wavelength autofluorescence (**A**), near-infrared autofluorescence (**B**) and optical coherence tomography (OCT, **C**) of the proband showing horizontal linear branching pattern focal ellipsoid zone loss. The proband’s sister had normal autofluorescence (**D**, **E**) and OCT (**F**). The proband’s mother had punctate hyperautofluorescence in the fovea (**G**) and reduced foveal near-infrared autofluorescence (**H**) with irregular ellipsoid line (**I**)

Macular OCT showed focal ellipsoid zone disruption in the proband and hyperreflective subretinal deposits in proband (Fig. 2A and C) and his mother (Fig. 2G and I). There was stippled and linear hyperautofluorescence resembling a butterfly-shaped pigment dystrophy in the proband (Fig. 2A and B), whilst his mother had scattered hyperautofluorescent spots in the fovea (Fig. 2G). The proband’s sister had a normal macular OCT scan with normal outer retinal band thicknesses and normal FAF pattern (Fig. 2D–F). UWF FAF imaging demonstrated mild disturbance of the autofluorescence signal in the annular zone of hyperpigmentation seen on pseudocolor imaging in all three patients (Fig. 1). Peripheral OCT scan in the proband showed thinning of the outer retinal bands in the region of reticular pigmentation and temporal retinoschisis in both eyes (Supplementary Material S4). Baseline microperimetry in the proband at age 48 revealed a shallow paracentral scotoma which progressed in the right eye at two years of follow-up (Supplementary material S5). The proband’s sister had normal microperimetry exam at age 54 (Supplementary material S5). EBST was unremarkable for the proband and his mother. However, the proband’s sister had consistent far peripheral loss of sensitivity on both sides when tested two years apart (Supplementary material S6).

### Electrophysiology

**Fig. 3:**
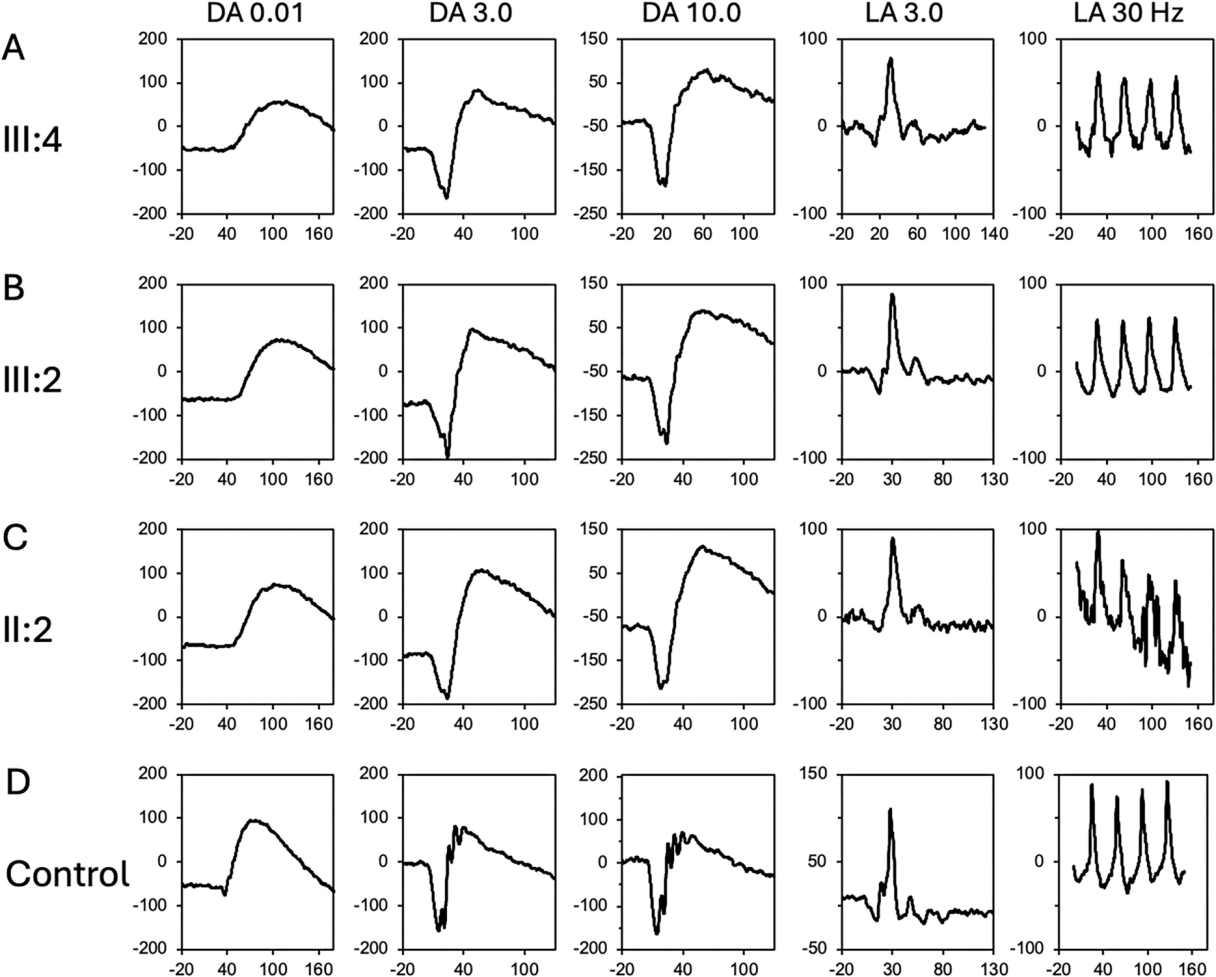
Full-field electrophysiology showing the proband (**A**), his sister (**B**), their mother (**C**) and a control (**D**). Dark-adapted (DA) 0.01 cd/m^2^ response showed delayed b-wave. The a-wave was reduced and delayed in DA 3.0 cd/m^2^ and DA 10.0 cd/m^2^. Light adapted 3.0 cd/m^2^ and 30 Hz flicker were within normal limits

**Fig. 4:**
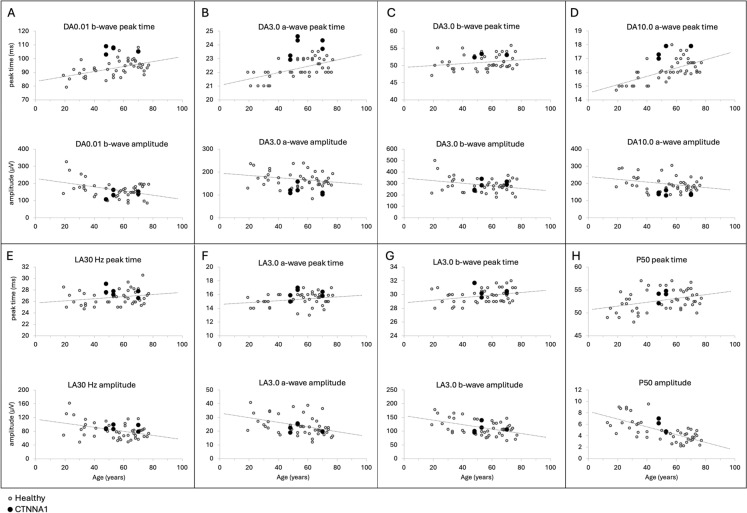
Scatter plots showing the three subjects’ recordings at age 48 (proband), 52 (proband’s sister) and 67 (proband’s mother) (solid circles) relative to normal controls (open circles). Dark-adapted (DA) 0.01 cd/m2 b-wave (**A**) and DA 3.0 cd/m2 a-wave (**B**) responses were delayed with subnormal amplitude. The b-wave parameters were within normal range in DA 3.0 cd m2 (**C**). DA 10.0 cd/m2 (**D**) also showed a delayed and subnormal a-wave. Light-adapted (LA) 30 Hz flicker (**E**) and 3.0 cd/m2 single flash responses (**F** and **G**) were within normal limits. Pattern electroretinography P50 component was within normal limits (**H**)

The light peak-to-dark trough (LP:DT) ratio was reduced to below 1.7 (normal range 1.7 to 4.3)[10] in one or both eyes in all three patients (Supplementary material S7). The predominant abnormality is seen in dark-adapted (DA) full-field ERG components (Fig. 3). The DA0.01 b-wave showed delay in III:4 and III:2. In both DA3.0 and DA10.0, there was also delay in a-wave peak time in III:2 and II:2 but marginal reduction in amplitude. The b:a ratio for DA3.0 and DA10.0 were within normal range. Light-adapted responses were all within normal range [12]. The proband’s multifocal ERG showed central macular dysfunction whilst his sister’s multifocal ERG revealed marginal response density reductions and implicit time delay in the outer ring (Supplementary material S8)[11]. Pattern ERG components were within normal limits for the proband and his sister [13]. A comparison of the ERG components compared to a cohort of healthy controls is shown in Fig. 4 demonstrating mild rod photoreceptor dysfunction with mild DA0.01 b-wave and DA3.0 a-wave delay above the 97.5 centile compared with age-appropriate normative values.

## Discussion

Seven pathogenic missense variants in *CTNNA1* (Glu307, Leu 318, Ser 322, Thr325, Ile431, Glu432 and Ser439) have been described by two groups. The p.(Leu318Ser) variant was identified as the cause of butterfly-shaped pigment dystrophy in a pedigree of nine affected individuals across two generations [6, 7]. The *CTNNA1*-related macular phenotype includes cases like a 7-year-old with a normal fundus, but non-penetrance of the typical macular phenotype has not been observed in an adult as demonstrated by III:2, whose multimodal imaging and microperimetry remained normal from ages 52 to 54. Despite no visible fundus lesions, her multifocal ERG revealed outer ring abnormalities. More severe macular phenotypes that result in visual loss include pigment epithelial detachment, subretinal fluid and extensive macular RPE atrophy. In the proband, we demonstrated that even butterfly-shaped pigment dystrophy may exhibit progressive paracentral scotoma enlargement on microperimetry over 32 months, consistent with multifocal ERG changes, despite stable visual acuity and no apparent OCT or FAF changes.

Peripheral retinal changes in *CTNNA1*-associated retinal dystrophy are not well characterized. Saksens et al. described three members of the p.(Leu318Ser) family exhibiting peripheral bone spicule-like lesions without supporting clinical illustration [7]. Tanner et al. described two cases: p.(Glu432Lys) with reticular pigmentary changes initially mistaken as retinitis pigmentosa and p.(Ser439Phe) with peripheral scalloped atrophic areas [8]. Full-field ERG was normal in these five cases. It remains unclear if UWF imaging was used to exclude peripheral changes in cases without peripheral lesions. Our findings highlight consistent peripheral reticular pigmentary changes in all three carriers of p.(Leu318Ser) with structural changes on peripheral OCT and its functional impact as measured on EBST and full-field ERG. Peripheral OCT revealed peripheral retinal thinning, and the proband’s sister showed persistent but non-progressive peripheral sensitivity loss over two years. All three cases exhibited mild rod photoreceptor dysfunction with borderline delayed b-wave (DA0.01) and mildly delayed a-wave (DA3, DA10), without affecting the cone system. While this subtle rod dysfunction did not result in nyctalopia, a mild dark-adaptation deficit cannot be excluded without a formal dark adaptometry.

The mild reduction in rod function does not explain the severely reduced EOG light-rise in our cases, which was reported in 72% of prior cases that had undergone an EOG. Differential diagnoses of reduced EOG light-rise include *OTX2*-associated retinal dystrophy [5], and autosomal dominant vitreoretinochoroidopathy (ADVIRC) due to selected bestrophin-1 (*BEST1*) variants [17]. *OTX2*-associated retinal dystrophy can also present with reduced EOG light rise [5]. However, the macular pattern dystrophy has a stellate-shaped border with shallow subretinal fluid, and there can be associated megalopapilla, multiple cilioretinal arteries and temporal peripheral avascular retina[18]. ADVIRC shares features of *CTNNA1* such as peripheral annular pigmentation and reduced EOG light rise. However, in ADVIRC, the peripheral pigmentary pattern is typically clumped, with a distinct posterior demarcation line [17]. *CTNNA1*-associated retinal dystrophy also lacks ADVIRC-associated features such as microcornea, shallow anterior chamber, iris dysgenesis, macular oedema, optic nerve dysplasia, progressive generalised atrophy and severe ERG abnormalities distinguishing it from *BEST1*-related ADVIRC [17, 19–23]. Both *CTNNA1* and *BEST1* are associated with generalised RPE dysfunction and macular and peripheral retinal changes. Clinically, *CTNNA1* should be considered in butterfly-shaped pigment dystrophy and without ellipsoid band thickening typical of *PRPH2* retinopathy [24]. The use of EOG to demonstrate generalised RPE dysfunction and the observation of peripheral reticular pigmentations aid in distinguishing *CTNNA1*.

Limitations of this study include a small sample size, short follow up and data from a single family. Nevertheless, we demonstrated variability in the macular phenotype and consistent peripheral reticular pigment changes which may be more common than previously reported. Detailed structure–function analysis demonstrated microperimetric progression of central macular dysfunction despite stable acuity and mild functional impact of the peripheral reticular pigmentary changes on EBST and full-field ERG. The increase in paracentral scotoma size with a lack of reduction in mean sensitivity on microperimetry suggests the importance of examining the sensitivity map for regional changes.

This study expands the *CTNNA1*-associated retinal dystrophy spectrum to include enlarging paracentral scotoma from butterfly-shaped pigment dystrophy and functionally significant peripheral reticular pigmentary changes. These features help refine differential diagnosis in patients with macular and peripheral pigmentation associated with reduced EOG light-rise. Further research to understand how these variants lead to RPE and rod photoreceptor dysfunction are critical for informing the development of effective therapeutic interventions for *CTNNA1*-associated retinal dystrophy.

## Supplementary Information

Below is the link to the electronic supplementary material.

Supplementary file1 (PDF 2905 kb)
