# Supplementary material for: CTNNA1-associated retinal dystrophy: novel multimodal imaging and electrophysiology features
Source: Doc Ophthalmol. 2025 Jun 2;151(3):239–46. doi: 10.1007/s10633-025-10027-0 (PMC12568884; doi:10.1007/s10633-025-10027-0)
Supplement: Supplementary file 1 — Supplementary file1 (PDF 2905 kb) [file 10633_2025_10027_MOESM1_ESM.pdf]

***CTNNA1*-associated retinal dystrophy: Novel multimodal imaging and electrophysiology features**

Jonathan A. Alexis, Prathiba Ramakrishnan, Matthew K. Kenworthy, Jennifer A. Thompson, Enid S. Chelva, Fred K. Chen

Supplementary Materials

S1: A table of demographic and phenotypic features of published *CTNNA1* cases

S2: Pedigree

S3: A table summarising clinical and multimodal imaging features

S4: Peripheral OCT in the Proband

S5: Microperimetry in proband and his sister

S6: Esterman binocular suprathreshold test

S7: Electro-oculography

S8: Multifocal electroretinography

Supplementary Material S1: A table of demographic and phenotypic features of published *CTNNA1* cases

Variant	Family	Individual	Age	Sex	Visual Acuity (at initial presentation)		Macular Lesion	Peripheral Lesion	EOG		ERG		Follow up duration and outcome
					OD	OS			OD	OS	OD	OS	
c.919G>A p.(Glu307Lys)	Family C†	C-II:1	33	F	20/100	20/100	Starfish-like pattern dystrophy in OU with pigmented subretinal deposits radiating outwards from denser deposits in foveal area; small, whitish dots on outline of 6 starfish legs; highlighting of macular luteal pigment, more pronounced in central foveal area, pigment epithelial detachment OD	None described	1.7	1.4	Normal	Normal	11 years
		C-I:2	59	F	20/25	20/20	Mild pattern dystrophy OU with two (OD) and three (OS) loosely defined linear tracts containing irregular patches of hyperpigmentation radiating from denser central foveal lesion	None described	1.8	1.7	-	-	Unknown
		C-III:1	7	F	20/25	20/20	Normal fundus OU	Normal fundus OU	-	-	Normal	Normal	Unknown
c.953T>C p.(Leu318Ser)	Family A†	A-II:1	70	M	20/20	20/20	Parafoveal chorioretinal atrophy OU	Peripheral bone spicule-like structures	1.6	1.5	Normal	Normal	27 years
		A-II:3	68	M	20/20	20/25	Typical butterfly retinopathy in center of macula (OU)	Extensive bone spicule like peripheral hyperpigmentation	1.0	1.0	Normal	Normal	26 years
		A-II:5	71	M	20/20	20/20	General hypopigmentation with central RPE atrophy	Bone spicule-like pigmentations in peripheral retina	1.3	1.3	Normal	Normal	26 years
		A-II:8	66	M	20/50	20/200	Typical butterfly retinopathy in center of macula with central chorioretinal atrophy OU	None described	1.9	2.0	Normal	Normal	15 years
		A-III:2	50	M	20/32	20/40	Butterfly-shaped pigmented lesion macula OU	No peripheral abnormalities	1.4	1.5	Normal	Normal	36 years
		A-III:7	40	F	20/40	20/16	Typical butterfly-shaped hyperpigmentation surrounded by hypopigmentation OD. No abnormalities in OS	None described	1.4	1.4	-	-	Unknown
		A-III:10	35	M	20/20	20/40	Butterfly-shaped hyperpigmentation OU	None described	1.4	1.5	-	-	3 years
		A-III:11	36	M	20/25	20/20	Typical butterfly-shaped hyperpigmentation surrounded by hypopigmentation OD and a juxtafoveal area of hyperpigmentation surrounded by a small ring of hypopigmentation OS	None described	3.0	2.7	-	-	1 year
		A-III:12	26	F	20/25	20/25	Typical butterfly-shaped hyperpigmentation surrounded by hypopigmentation OS. Small pigmentary changes without any pattern OD	None described	1.8	1.7	-	-	1 year
	**Current Study**	**II:2**	**67**	**F**	**20/25**	**20/20**	**Butterfly-shaped pigment dystrophy OU**	**Peripheral, annular reticular hyperpigmentation and retinoschisis OU**	**1.6**	**1.9**	**Abnormal**	**Abnormal**	**2 years, no change**
		**III:2**	**52**	**F**	**20/20**	**20/16**	**No abnormality OU**	**Peripheral, annular reticular hyperpigmentation OU**	**1.5**	**1.6**	**Abnormal**	**Abnormal**	**4 years, no change**
		**III:4**	**48**	**M**	**20/20**	**20/25**	**Pigmentary change in fovea OU**	**Peripheral, annular reticular hyperpigmentation OU**	**1.2**	**1.3**	**Abnormal**	**Abnormal**	**2 years, no change**

c.965C>T p.(Ser322Leu)	Family 1✦	Proband	43	F	20/20	20/17	Macular pigmentary clumping OU	Peripheral reticular changes	1.5	1.5	Normal	Normal	5 months, VA 20/30 OU
		Daughter	4	F	20/40	20/40	Central foveal deposit OU	None described	-	-	Normal	Normal	3 months, VA 20/40 OU
		Daughter	19	F	20/20	20/20	Subtle central altered foveal reflex OU	None described	1.7	1.65	Normal	Normal	Unknown
	Family 3✦	Proband	27	F	20/20	20/20	Pigmentary clumping OU	None described	-	-	Normal	Normal	21 months, no change
		Mother	63	F	-	-	Subtle pigmentary change OU	Peripheral drusen	-	-	-	-	Unknown
	Upadhyaya et al. case report	Proband	40	F	-	-	Butterfly-shaped pigmented lesion macula OU and neurosensory foveal detachment OU	None described	1.6	1.5	-	-	Unknown
c.973A>G p.(Thr325Ala)	Family 6✦	Proband	62	M	20/200	20/30	Central atrophy in OD and pigment mottling in OS (later becoming atrophy)	None described	2.6	2.0	Normal	Normal	17 years, VA 20/80 OD, 20/200 OS, marked macular atrophy OU
c.1293T>G p.(Ile431Met)	Family B†	B-I:2	71	F	20/200	20/63	Mild pattern dystrophy OU with one central lesion (OS) and several 'dot and halo' lesions (OD) in the macula	None described	-	-	-	-	Unknown
		B-II:1	62	M	20/20	20/25	OU atrophy of RPE and photoreceptors in the macula, with a less peculiar butterfly-shaped pattern of pigmentary changes OD and focal hyperpigmentation centrally OS	None described	-	-	-	-	7 years
c.1294G>A p.(Glu432Lys)	Family 5✦	Proband	31	F	20/20	20/20	Linear areas of pigmentary change	Peripheral retinal reticular pigmentary changes mistaken as retinitis pigmentosa	-	-	Normal	Normal	4 years, VA 20/17 OU, stable imaging, AF abnormalities more evident
c.1316C>T p.(Ser439Phe)	Family 2✦	Proband	43	M	20/17	20/17	Linear areas of hyper and hypo-pigmentation radiating from foveal centre	None described	1.35	1.45	Normal	Normal	18 years, VA 20/60 OD, 20/30 OS
		Sister	38	F	20/20	20/20	Subtle pigmentary changes	Peripheral areas of scalloped atrophic change (no image to show this)	1.45	1.45	Normal	Normal	11 months, no change
	Family 4✦	Proband	32	F	20/30	20/17	Linear areas of pigmentary change	None described	1.25	1.35	Normal	Normal	10 months, no change
		Maternal cousin	34	M	20/30	20/20	Linear areas of pigmentary change	None described	-	-	Normal	Normal	19 months, VA 20/ 20 OD, 20/17 OS

OD - right eye; OS - left eye; OU - both eyes; VA - visual acuity; EOG - electro-oculography; ERG – electroretinography,

† - Saksens et al; ✦ - Tanner et al.

Supplementary Material S2: Pedigree

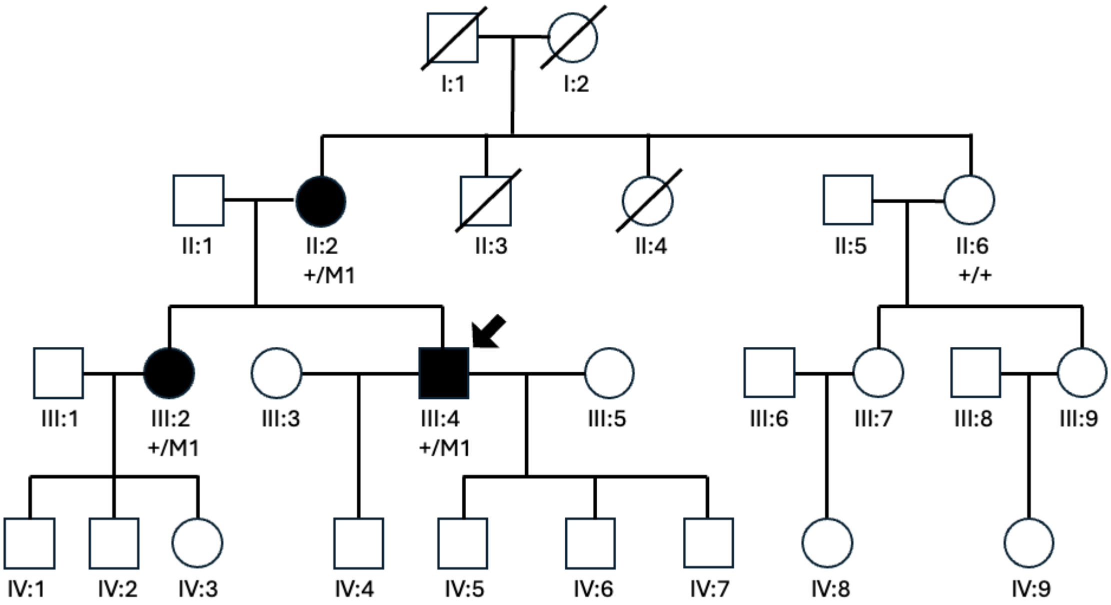

M1: c.953T>C; p.Leu318Ser

Arrow shows the proband

Supplementary Material S3: A table summarising clinical and multimodal imaging features

	Proband (III:4)	Sister (III:2)	Mother (II:2)
**Age (years) (initial → final)**	48 → 51	52 → 54	67 → 72
**Follow-up duration (months)**	32	26	49
**Sex**	M	F	F
**Best-corrected Visual acuity**	OD 20/20, OS 20/25 #	OD 20/20, OS 20/16	OD 20/25, OS 20/20
**Refraction**	N/A	OD -2.75 / -1.25 x 78<br>OS -3.25 / -0.75 x 95	OD -0.25 / -0.25 x 76<br>OS -0.50 / -0.50 x 48
**Symptom**	White dots on eccentric gaze	Asymptomatic	Asymptomatic
**Anterior segment**	OS posterior subcapsular cataract	Normal	Normal
**Posterior segment**	Foveal butterfly-shaped pigmentation OU<br>Peripheral, annular reticular pigmentation, Temporal retinoschisis OU	Normal macula OU<br>Peripheral, annular reticular pigmentation OU	Foveal pigmentation OU<br>Peripheral, annular reticular pigmentation OU
**Axial length (mm)**	N/A	OD 24.79, OS 24.65	OD 23.71, OS 23.70
**OCT**	Outer retinal disruption and hyper-reflective subretinal deposits OU	Normal	Hyper-reflective subretinal deposits OU
**FAF**	Patches of hypoAF and linear hyperAF OU	Normal	Multifocal perifoveal hyperAF spots OU
**MM**	Progressive enlargement of paracentral scotoma	Normal	N/A
**EBST**	Normal	Peripheral losses	Normal

OCT – optical coherence tomography, FAF – fundus autofluorescence, OU – both eyes, OD – right eye, OS – left eye, AF – autofluorescence, EBST – Esterman binocular suprathreshold test, MM – MAIA microperimetry, NA – not available

uncorrected visual acuity is the same as best-corrected

Supplementary Material S4: Peripheral OCT in the proband

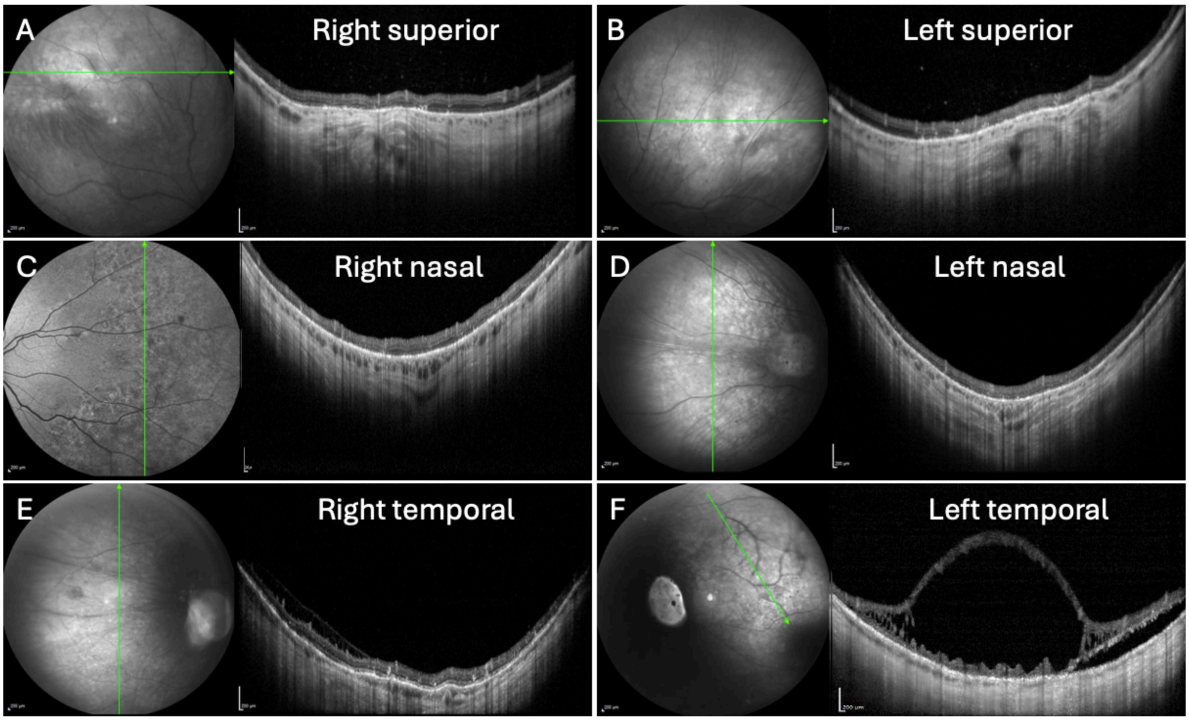

Single line superior horizontal optical coherence tomograph (OCT) scans showed loss of outer retinal layer structures in the region of reticular pigmentation in right (A) and left (B) eyes. Whorl-like scleral structures and choroidal thinning are also present. Single line nasal vertical OCT scan shows similar loss of outer retinal structures in the region of reticular pigmentation in right (C) and left (D) eyes. Superotemporal OCT scans show retinoschisis in the right (E) and left (F) eyes with localised subretinal fluid due to an outer leaf break.

Supplementary Material S5: Microperimetry in proband and his sister

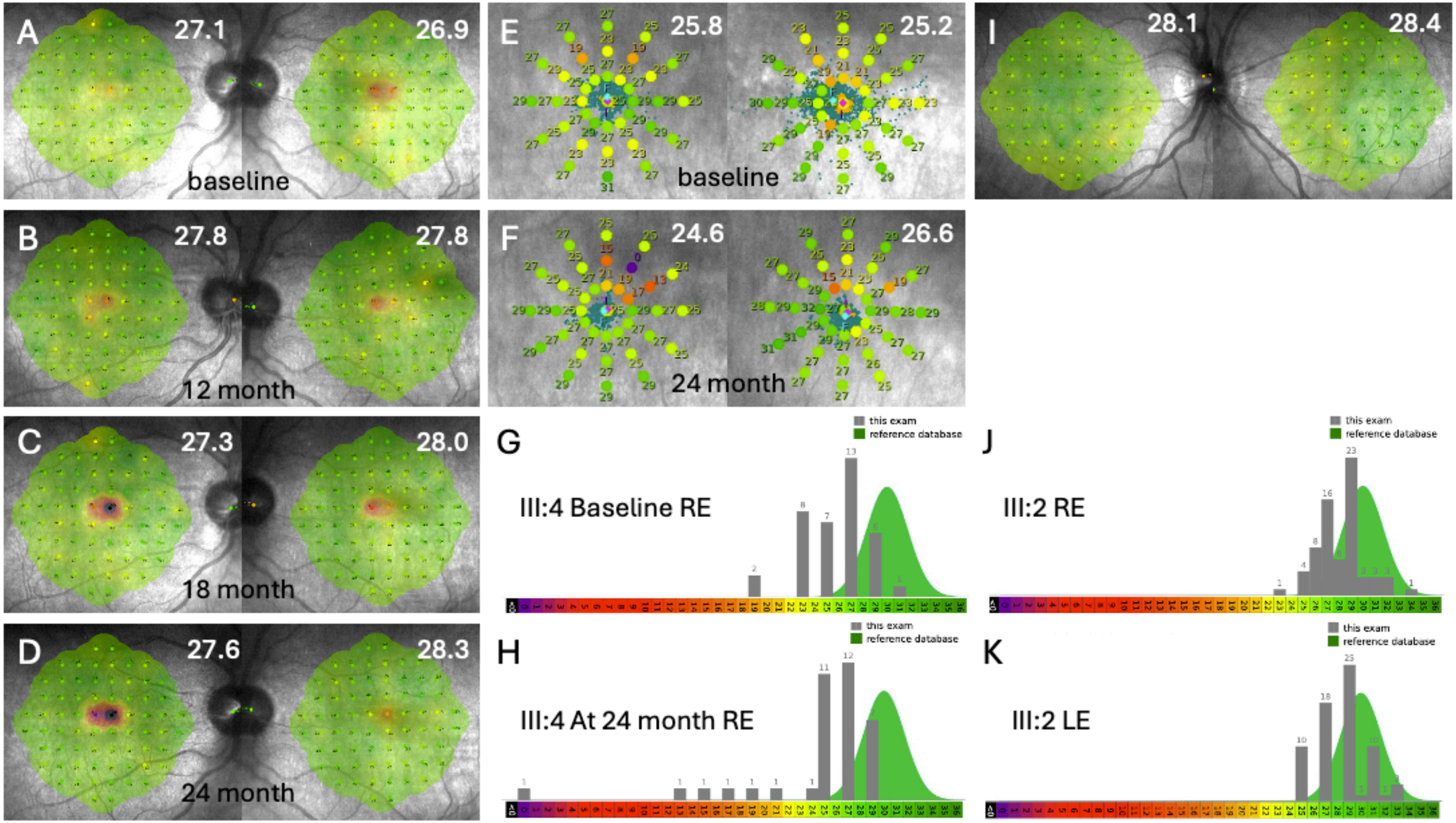

MAIA microperimetry interpolated maps of proband (III:4) at baseline (A), 12 months (B), 18 months (C) and 24 months (D) showing evolution of the paracentral scotoma superior to fovea in the right eye and stable scotoma in the left eye. Mean sensitivity values are shown in top right corner in dB. Small testing grid at baseline (E) and at 24 months (F) shows deepening of the scotoma at 1-2 degrees of eccentricity superonasal to fixation in the right eye and at 1 degree eccentricity superonasal to fixation in the left eye. Histogram of the threshold frequencies in right eye at baseline (G) and 24 months (H) showing a shift in distribution towards lower sensitivity. MAIA microperimetry interpolated map of the proband's sister (III:2) at age 54 (I) with normal histogram of the threshold frequencies in right (J) and left (K) eyes. RE: right eye; LE: left eye.

Supplementary Material S6: Esterman binocular suprathreshold test

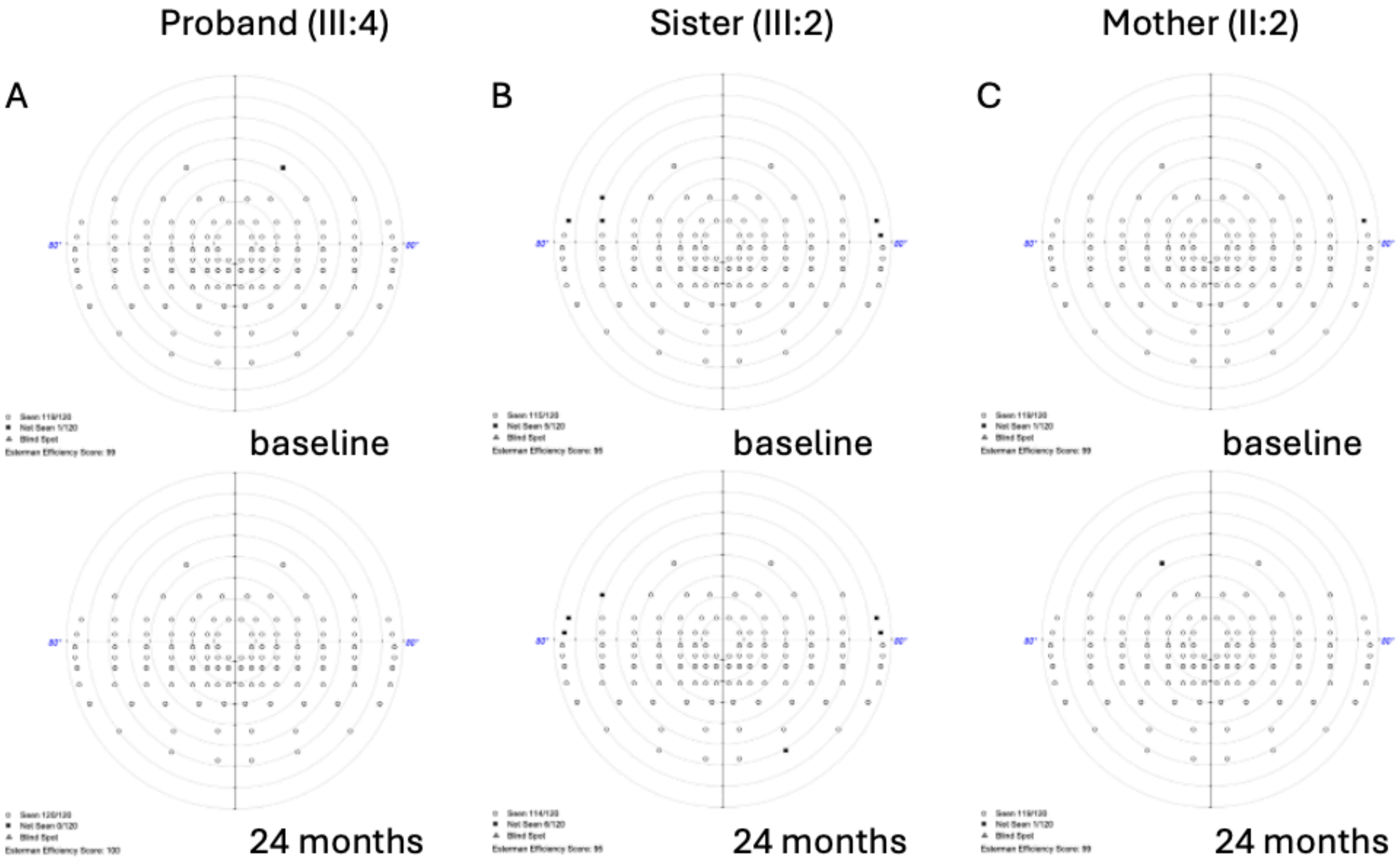

Esterman binocular suprathreshold test showed normal responses in the proband (A) 2 years apart. In his sister, there was persistent peripheral loss of sensitivity reproducible at two years later (B). His mother had normal threshold responses (C) 2 years apart.

Supplementary Material S7: Electro-oculography

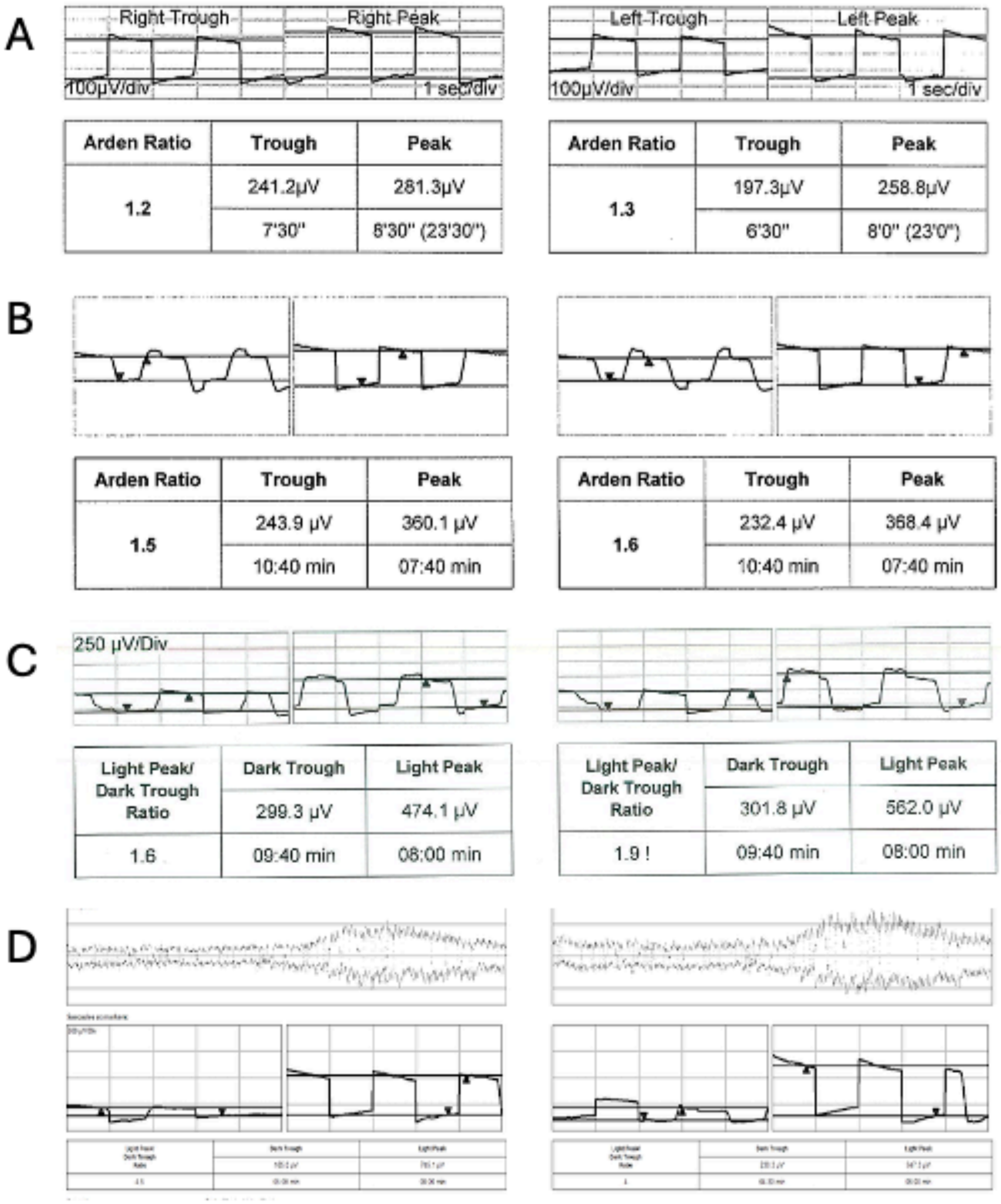

Electro-oculography for proband (A), his sister (B), their mother (C) showing light peak to dark trough ratios for both eyes compared to a healthy control (D).

Supplementary Material S8: Multifocal electroretinography

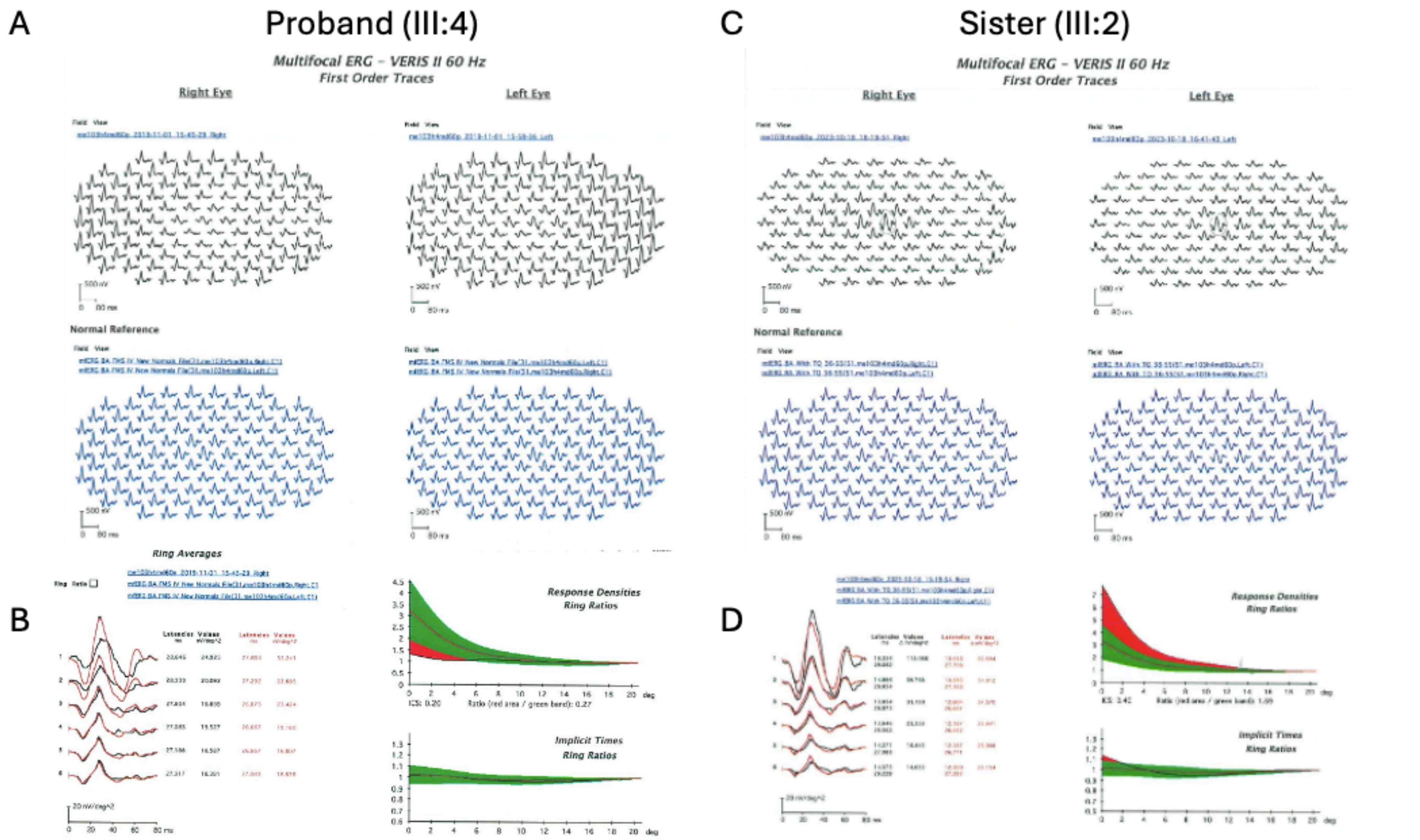

Multifocal electroretinography (mfERG) trace array of the proband (A) showing central macular response density loss in both eyes (A) and reduced ring ratio for the central hexagons (B). Multifocal ERG trace array of the proband's sister (C) showing reduced response density in the peripheral macula resulting in increased ring ratio for the central hexagon (D).
